# TME13/452: Internet Facilitated Pediatric Care: Nocturnal enuresis

**DOI:** 10.2196/jmir.1.suppl1.e120

**Published:** 1999-09-19

**Authors:** R Pretlow

**Affiliations:** ^1^eHealth International, KirklandUSA

**Keywords:** Enuresis, Bedwetting, Internet

## Abstract

**Introduction:**

Internet health care applications appear to be centering on three areas: 1) disease monitoring and management, 2) patient education, and 3) patient support groups. This presentation demonstrates how Internet technology can be advantageous in managing certain childhood disorders, as an example, nocturnal enuresis. Treatment options for nocturnal enuresis currently include behavioral modification, medications, wetness alarms, and bladder volume alarms. Regardless of which treatment modality is chosen, considerable time is required on the part of the health care provider to educate, manage, and support the child and parent. Moreover, the impact of enuresis on a child's self-esteem can be profound. Enuresis tends to be a "closet" malady, often leaving the child feeling isolated and "different." To date, support groups for enuresis are rare. A need therefore exists to combat this social isolation and self-esteem impact.

**Methods:**

Internet technology offers potential solutions to all of the above issues. The author has designed a pilot Web site specifically for childhood enuresis. The site contains separate areas for children and for teenagers, tailored to the age of the patient, and an area for parents. Each area has the same basic functions of: 1) interactive enuresis management with the patient's health care provider, 2) patient education, and 3) interactive message boards and chat rooms for social support. Interactive management with the health care provider is accomplished via auto-uploaded "secure" patient data and via password-protected patient "rooms" to which provider reports and instructions are downloaded. Educational content includes case histories, anatomy and physiology lessons, explanations of current treatments, and explanations of the latest enuresis research. The "threaded" message board and chat room functions allow anonymous interaction with other enuretic children of the same age, as well as parental interaction with other parents.

**Results:**

The enuresis Web site has been well received by a pilot group of 10 enuretic children and their parents. Variables such as treatment response, parental satisfaction, and patient self-esteem appear significantly improved, in this uncontrolled study.

**Discussion:**

The management of nocturnal enuresis can be facilitated using Internet technology. Other chronic pediatric disorders such as diabetes, asthma, allergies, behavioral problems such as attention deficit disorder, learning disabilities, growth disorders, and congenital defects appear to be candidates for similar interactive Internet management. Additionally, well-child care could be facilitated, especially in remote areas.

